# Cuproptosis-related genes score: A predictor for hepatocellular carcinoma prognosis, immunotherapy efficacy, and metabolic reprogramming

**DOI:** 10.3389/fonc.2023.1096351

**Published:** 2023-02-09

**Authors:** Guilin Nie, Dingzhong Peng, Ningyuan Wen, Yaoqun Wang, Jiong Lu, Bei Li

**Affiliations:** ^1^ Department of Biliary Surgery, West China Hospital of Sichuan University, Chengdu, China; ^2^ Department of General Surgury, Division of Hepatobiliopancreatic Surgery, Nanfang Hospital, Southern Medical University, Guangzhou, Guangdong, China; ^3^ Research Center for Biliary Diseases, West China Hospital, Sichuan University, Chengdu, Sichuan, China

**Keywords:** cuproptosis, hepatocellular carcinoma, prognostic model, sorafenib, GLS

## Abstract

**Background:**

Cuproptosis is a newly identified type of programmed cell death, characterized by aggregation of mitochondrial lipoylated proteins and the destabilization of Fe–S cluster proteins triggered by copper. However, its role in hepatocellular carcinoma (HCC) remains unclear.

**Methods:**

We analyzed the expression and prognostic significance of cuproptosis-related genes using the data obtained from TCGA and ICGC datasets. A cuproptosis-related genes (CRG) score was constructed and validated *via* least absolute shrinkage and selection operator (LASSO) Cox regression, multivariate Cox regression and nomogram model. The metabolic features, immune profile and therapy guidance of CRG-classified HCC patients were processed *via* R packages. The role of kidney-type glutaminase (GLS) in cuproptosis and sorafenib treatment has been confirmed *via* GLS knockdown.

**Results:**

The CRG score and its nomogram model performed well in predicting prognosis of HCC patients based on the TCGA cohort (training set), ICGC cohort and GEO cohort (validation set). The risk score was proved as an independent predictor for overall survival (OS) of HCC. The area under the curves (AUCs) of the model in the training and validation cohorts were all around 0.83 (TCGA, 1- year), 0.73 (TCGA, 3- year), 0.92 (ICGC, 1- year), 0.75 (ICGC, 3- year), 0.77 (GEO, 1- year), 0.76(GEO, 3- year). Expression levels of metabolic genes and subtypes of immune cells, and sorafenib sensitiveness varied significantly between the high-CRG group and low-CRG group. One of the model-included gene, GLS, might be involved in the process of cuproptosis and sorafenib treatment in HCC cell line.

**Conclusion:**

The five cuproptosis-related genes model contributed to prognostic prediction and provided a new sight for cuproptosis-related therapy in HCC.

## Introduction

Several types of regulated programmed cell death have been reported, including apoptosis, pyroptosis, necroptosis, and ferroptosis (1). Ferroptosis, a unique modality of cell death driven by iron-dependent phospholipid peroxidation, is regulated by complex cellular metabolic events, including redox homeostasis, iron handling, mitochondrial activity, and amino acids metabolism, lipid metabolism, sugar metabolism, in addition to numerous signaling pathways relevant to disease (2–4). Similar to iron, copper is a cofactor for essential enzymes and has been recognized to induce the aggregation of lipoylated dihydrolipoamide S-acetyltransferase (DLAT) caused by FDX1. Lipoylated DLAT is associated with the mitochondrial tricarboxylic acid (TCA) cycle, resulting in proteotoxic stress and a novel form of cell death called “cuproptosis” (5). In brief, the copper-induced death was independent of known cell death pathway and relied on mitochondrial metabolism. The new mechanistic information suggests that other trace mentalion-induced forms of cell deaths exist (6). Similar to ferroptosis induction-based cancer therapies in nanoparticle-based gene-target therapies or in combination with other therapeutic approaches (7), curoptosis induction-based treatment provides new therapeutic approaches for cancers with the development of curoptosis-related research.

The role of copper in the development of HCC remains unknown. Otherwise, some studies have proved that copper contributes to hepatocarcinogenesis and its progression. Copper contributes to glycolytic metabolism and the tumorigenic properties of hepatocellular carcinoma (HCC) (8). A copper complex, [Cu(ttpy-tpp)Br^2^]Br (referred to as CTB), could induce HCC cells senescence and act as an antitumor compound *via* SLC25A26 and methionine cycle (9). Disulfiram combined with copper inhibits metastasis and epithelial-mesenchymal transition in HCC *via* NF-κB and TGF-β pathways (10). The copper metabolism MURR1 domain 10 (COMMD10) increased intracellular Cu and led to radioresistance of HCC (11). In addition, the increased incidence of HCC in patients and animal models with Wilson disease was found to be associtated with an unknown mechanism promoting the malignant process resulting from copper accumulation (12). The emergence of cuproptosis provides new insights into copper-based therapies for HCC.

This study explored the mechanism of copper-induced cell death underlying HCC. We rescreened the genes involved in copper ionophore-induced death. The data was acquired from the genome-wide CRISPR-Cas9 loss-of-function screens dataset provided by Peter Tsvetkov (5). The association between cuproptosis-related genes and HCC prognosis was explored and the genes were used to establish a prognosis-predicted model, which demonstrated potential in grading HCC in terms of copper-induced cell death.

## Materials and methods

### Public data acquisition and processing

The cuproptosis-related genes datasets were acquired from the Tsvetkov’s study. The gene expression data, phenotype data, and corresponding survival information (if available) of HCC of The Cancer Genome Atlas (TCGA), the liver cancer project (code: LIRI_JP) of the International Cancer Genome Consortium (ICGC), and GSE14520 were downloaded from public databases. The single-cell analysis of GLS, FDX1, and CDKN2A in HCC was conducted in the Tumor Immune Single-Cell Hub (TISCH) database (http://tisch.comp-genomics.org/ ) and used the data from GSE125449. Additional ethical approval was not required since these data were all publicly available online.

### Construction and validation of a cuproptosis-related gene model

OVISE cells were used to screen cuproptosis-related genes. The OVISE-Cas9 cells were then infected with Brunello virus obtained from the Brunello virus library, which contains 76411 sgRNAs targeting 19114 genes. After the primary treatment of 50nM elesclomol-Cu, 200nM Cupric diethyldithiocarbamate (Cu-DDC), or DMSO control to the final dose of 250nM elesclomol-Cu and 700nM Cu-DDC, genomic DNA was isolated and sequenced by the Broad Genetic Perturbation Platform and Broad Genomics Platform for the establishment of gene library (5). The different expression genes were computed and considered as cuproptosis-related genes with an FDR < 0.05. 27 genes were possibly involved in copper ionophore-induced cell death and 25 genes expression levels (AFG3L2, AHR, BRPF1, CAPRIN1, CDKN2A, COQ7, DLAT, DLD, EGLN1, FDX1, GLS, HAUS5, LIAS, LIPT1, MBTPS1, MTF1, OXA1L, PDHA1, PDHB, REXO2, RPL3, SCAP, SLFN11, SOX2, YEATS20) except MCUR1 and MPC1 were detected in the TCGA dataset and put into the LASSO regression *via* the algorithm. We utilized the “glmnet” package (version 2.0-16) to fit the logistic LASSO regression. The optimal penalty coefficient (λ = 0.00542) in the LASSO regression was identified with the minimum criterion and five genes (FDX1, GLS, CDKN2A, DLAT, and LIAS) were found out as independent risk factors for HCC patients. Ten-fold cross-validation was used to select the penalty term, λ. The binomial deviance was computed for the test data as the measure of the predictive performance of the fitted models. The standard errors of the LASSO coefficients were obtained *via* bootstrapping within the primary sampling unit and strata (13). To performed the HRs of related genes, we showed totally ten HRs of genes included in the LASSO regression.

## Gene signature model construction and validation of predictive nomogram

Nomograms are widely used to predict the survival probability of cancer. Clinical characteristic parameters and prognostic signature were adopted to establish a nomogram to quantitatively investigate the probability of 1-, 3-, and 5-year survival of HCC patients. To construct the nomogram, multivariate regression analysis was applied to select the significant predictors of mortality. The nomogram was trained using the TCGA data and externally validated using the ICGC data. Discriminative performance was measured by concordance index (C-index). Calibration plots were used to describe the degree of fit between actual and nomogram-predicted mortality (14).

## Enrichment analysis

Gene ontology (GO) annotation and Kyoto Encyclopedia of Genes and Genomes (KEGG) pathway enrichment analyses between the two groups of patients were performed with the ‘clusterProfiler’ R package. An FDR <0.05 and |log2FC| ≥1 was set as the cut-off values.

## Immune profile analysis

The immune score and stromal score of each sample in the TCGA-LIHC cohort were calculated by the “estimate” package in R. The proportion of immune cells in the tumor microenvironment (TME) of each sample was evaluated *via* the TIMER, CIBERSORT, and CIBERSORT-ABS algorithm in R software. The single-sample gene set enrichment analysis (ssGSEA) with R package GSVA was used to elevate the degree of 16 immune cells infiltration and the activity level of 20 immune-related functions in HCC samples. The differential expression of immune checkpoints between risk groups was analyzed *via* R package limma.

## *In vitro* cell culture, maintenance, and transfection

The human HCC cell lines, PLC/PRF/5, and Huh7 were a gift from Professor Peng Yong (State Key Laboratory of Biotherapy, Sichuan University). Both cell lines were incubated at 37°C with 5% CO2 in Dulbecco’s Modified Eagle’s Media (DMEM) (Gibco BRL, Grand Island, NY, USA) containing 10% FBS (Gibco, Australia). The cells were transfected with si-RNAs using Lipofectamine™ 3000 (Thermo Fisher Scientific, USA) in accordance with the manufacturer’s instructions.

## RNA preparation, reverse transcription, and quantitative real-time PCR analysis

Total RNA was extracted using TRIzol Reagent (Invitrogen, Carlsbad, USA), and the concentration and purity of total RNA were determined by Nanodrop 2000 spectrophotometry (Thermo Scientific, USA). Reverse transcriptions were performed using Superscript III transcriptase (Invitrogen, Grand Island, NY). The cDNA amplification was performed using SYBR Green Real-time PCR Master Mix (Roche, Mannheim, Germany). 18S was used as the internal reference gene. The results were analyzed by the 2^-ΔΔCt^ method. The primer and si-RNA sequences were as follows: 5’-TTCCAGAAGGCACAGACATGGTTG-3’ (forward) and 5’-GCCAGTGTCGCAGCCATCAC-3’ (reverse) for GLS, and si-GLS: 5’-GAUGGACAGAGGCAUUCUA-3’ (sense).

## Western blot analysis

Protein was extracted from HCC cells using RIPA lysis buffer and quantified using a BCA kit (Thermo, USA). The protein samples were separated by 10% SDS–PAGE and transferred to PVDF membranes (Millipore, USA). After blocking for 1 h in 5% skim milk powder at room temperature, the membranes were incubated with primary antibodies at 4°C overnight, followed by an incubation with an anti-rabbit secondary antibody (1:10000, Abcam, UK) and visualized using Immobilon™ Western Chemiluminescent HRP Substrate (Millipore, USA).

## CCK8 assay

The effects of drugs and Copper on HCC cell lines were measured using CCK8 assay. Cells were seeded in 96-well plates at a density of 5 × 10^3^ cells/well, cultured overnight, and subsequently treated with various concentrations of drugs or DMSO. A medium containing CCK8 reagent was added after exposing the cells to drugs, and the optical density (OD) value at 450 nm of each well was measured. Sorafenib was purchased from Selleck Chemicals (USA). Elesclomol was obtained from MCE (USA), CuCl_2_ was from RHAWN (China).

## Immunohistochemistry analysis

The protein expression levels of the selected genes in normal and tumor tissues were confirmed by the Human Protein Atlas database (HPA; https://www.proteinatlas.org/). The relative expression levels of GLS were measured *via* Image J (http://rsb.info.nih.gov ).

## Statistical analysis

R software (version 4.2.1) with the “ggplot2,” “ggforest,” “cowplot,” “plot3D,” “maftools,” “VennDiagram,” “survminer”, “timeROC”, and “ggplotify” packages, and GraphPad Prism (version 9.0) were used for statistical analysis and data visualization. For comparison between the two groups in the bioinformatics analysis section, the Wilcoxon test was used for difference analysis to compare the risk scores of the two groups. The Students’-test was used to compare between the two groups in the experimental section like PCR or IHC. Two-way ANOVA was used for the results of CCK. As for more than two groups, the Kruskal-Wallis test was used, like comparing the risk scores of the different pathological stages. Kaplan-Meier survival analysis and log-rank tests were used the survival analysis of the different groups of patients. The univariate and multivariate Cox regression with calculation of hazard ratios (HR) and 95% confidence intervals (CI) was used to evaluate the importance of each parameter to OS. The t-ROC analysis measured the predictive power of the risk model and the predictive power was showed as the area under the curve (AUC). A two-tailed p <0.05 was considered statistically significant.

## Results

### FDX1, GLS, CKKN2A, DLAT, and LIAS were independent prognostic factors for HCC patients

**Figure 1:**
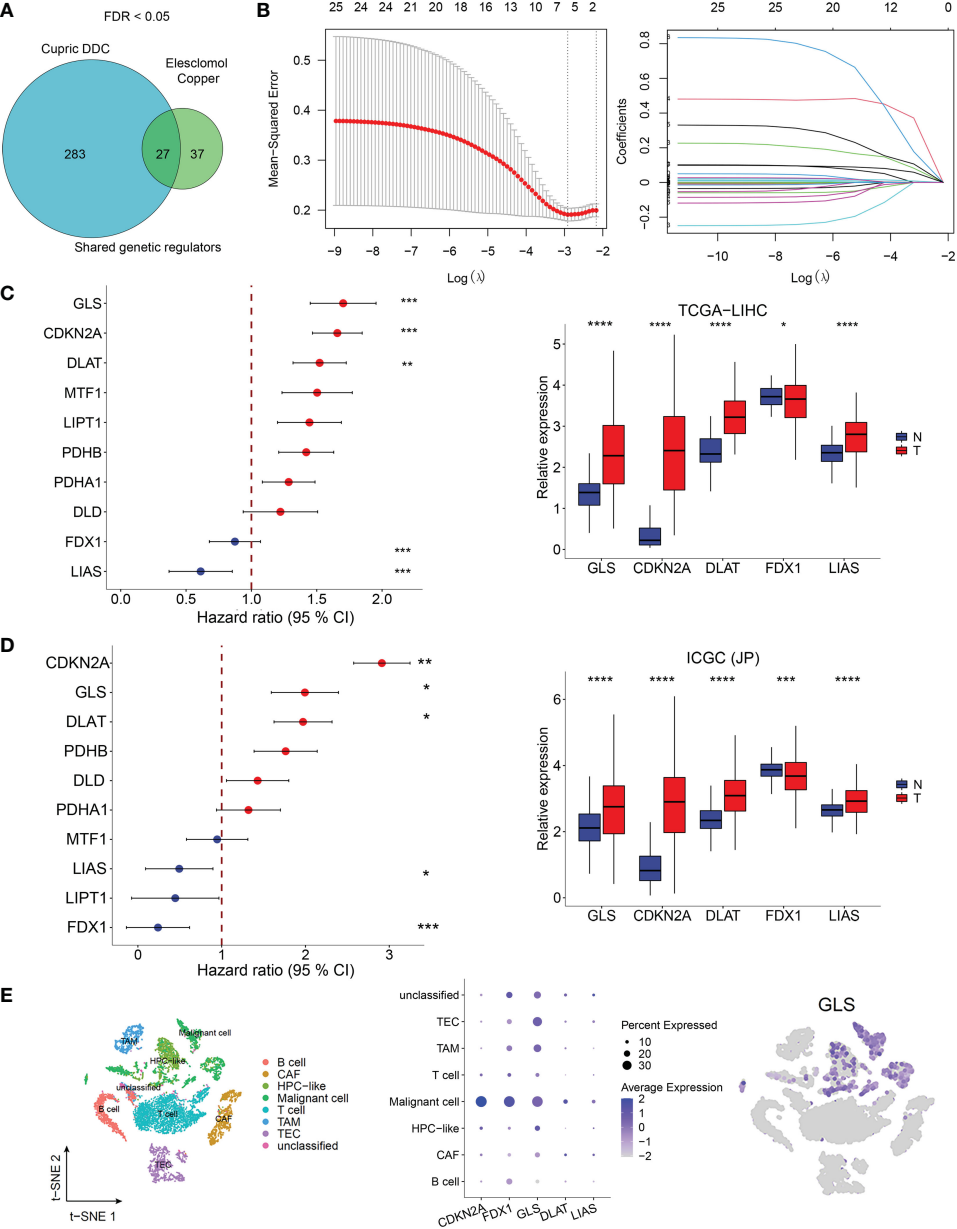
Cuproptosis-related genes screening in HCC. **(A)** Identification of cuproptosis-related genes. Whole-genome CRIPSR-Cas9 positive selection screen using two copper ionophores (Cu-DDC and elesclomol-copper). FDR < 0.05. **(B)** Tuning parameter (λ) selection by LASSO Cox regression (left). LASSO coefficient profiles of candidate gene expression (right). Multivariate Cox regression analysis used to screen the independent prognostic genes and relative expression of five genes in normol tissues and HCC tissues in TCGA dataset **(C)** and in ICGC dataset **(D, E)**The expression of CRG-included genes in eight types of cells in the GSE125449 dataset. The left t-SNE subgraph reveals the distribution of eight types of cells from HCC patients (distinguished by colors). The middle showed the relative expression of five genes in eight types of cells. The right subgraph showed the expression of GLS in each cell.

From the whole-genome CRISPR-Cas9 positive selection screen using two copper ionophores (Cu-DDC and elesclomol-copper) (FDR < 0.05), we identified 27 genes possibly involved in copper ionophore-induced cell death (**Figure 1A**) and 25 genes were included in a LASSO regression model. The range of positive and negative standard deviations of log(λ) was identified in the cross-validation plot. A vertical line was drawn at the value selected *via* cross-validation analysis of 10-fold. The function of the analysis to select important variables develops with the increased degree of compression and decreased value of λ (13). The optimal penalty coefficient (λ = 0.00542) in the LASSO regression was identified with the minimum criterion (**Figure 1B**). The results of survival analysis obtained from TCGA and ICGC datasets showed that FDX1, GLS, CDKN2A, DLAT, and LIAS were independent factors for HCC patients and might contribute to predicting the prognosis of HCC. Meanwhile, they were differentially expressed in HCC tumor and normal tissues (**Figures 1C, D**). In addition, single-cell sequence indicated that all five genes were included in GSE125449 and were predominantly detected in malignant cells (**Figures 1E**, **S1**).

### High cuproptosis-related genes risk score predicted the poor outcome of HCC patients

**Figure 2:**
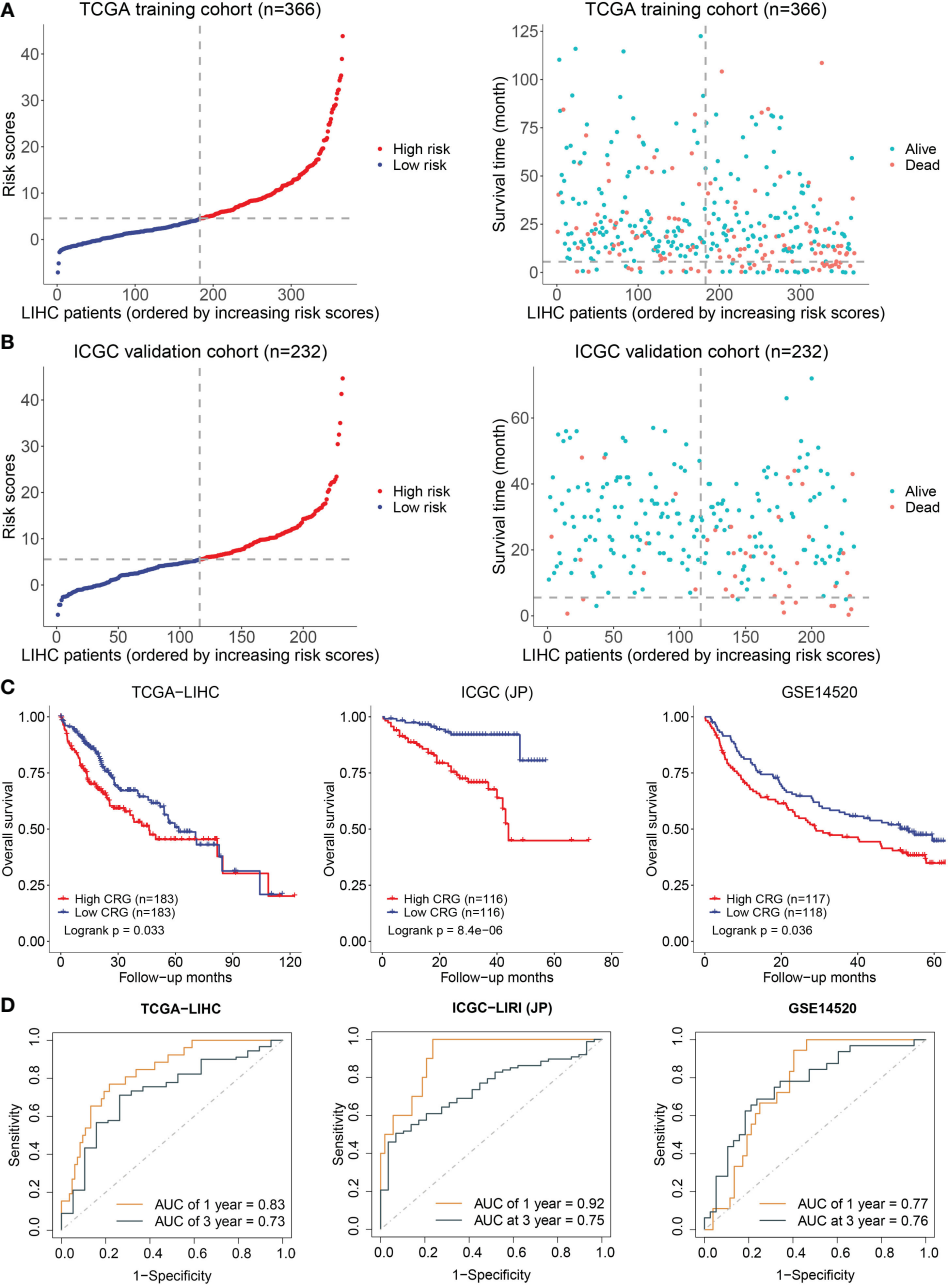
Establishment and validation of prediction model. **(A)** The distribution and optimal cutoff value of CRG (left), the OS status of each sample (right) according to TCGA dataset. **(B)** The CRG risk score status (left) and the OS status of each sample (right) according to ICGC dataset. **(C)** The Kaplan–Meier plots of CRG scores in the TCGA, ICGC, and GSE14520 cohorts based on the optimal cutoff value. **(D)** Time-dependent ROC analyses of the CRG for OS prediction in the TCGA, ICGC, and GSE14520 cohorts.

A prediction model was constructed based on the patients’ data from TCGA using a LASSO regression model and including the above five genes. The cuproptosis-related genes (CRG) risk scores = 0.53*GLS+0.42*DLAT+ 0.505*CDKN2A-0.135*FDX1-0.491*LIAS. The divided point of high risk and low risk was 4.6 (**Figure 2A**). The risk score distribution and outcome status showed that more deaths of HCC patients happened in higher risk score groups in our two cohorts (**Figure 2B**). The high-risk group had a shorter survival time than the low-risk group in the TCGA-LIHC dataset (training cohort, p = 0.033). Similar results were observed in the other two validation cohorts (p = 8.4x10^-06^ in ICGC-JP, p = 0.036 in GSE14520) (**Figure 2C**). The area under the curve (AUC) of the overall survival (OS) prediction model was 0.83 at one year and 0.73 at three years from TCGA. In the two external validation cohorts, the AUC values of the model in the ICGC-LIRI(JP) dataset were 0.92 at one year and 0.75 at three years, while they were 0.77 at one year and 0.76 at three years in the GSE14520 dataset (**Figure 2D**).

### Development and validation of a comprehensive nomogram based on clinicopathological characteristics and CRG risk score for clinic application

**Figure 3:**
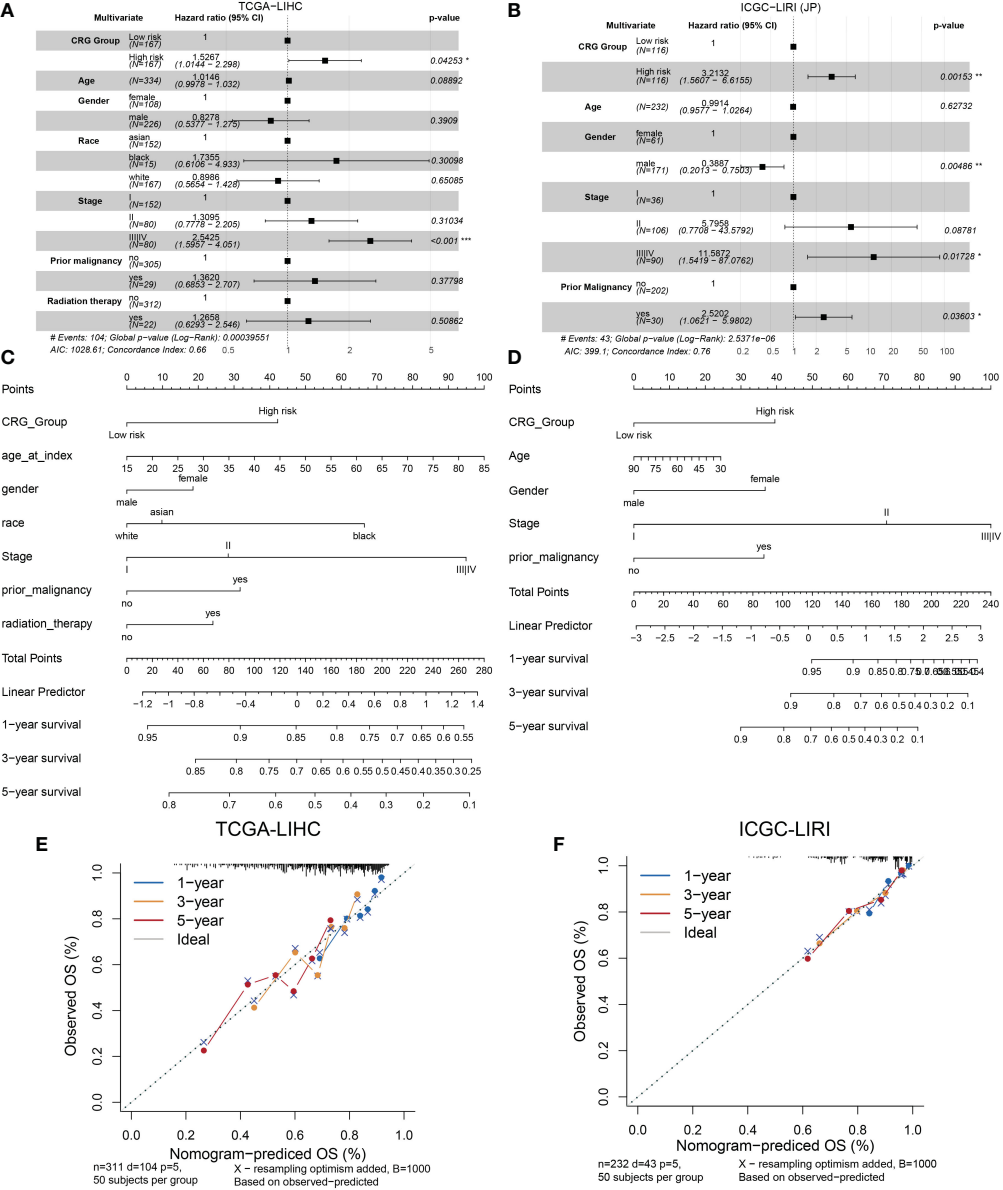
Establishment and validation of the model *via* nomogram. **(A)** Multivariate Cox regression analyses of CRG scores for OS in the TCGA cohort. **(B)** the ICGC cohort. **(C)** Nomogram of the clinical characteristic parameters and CRG scores for OS prediction according to the TCGA cohort. **(D)** the ICGC cohort. **(E)** Calibration plots according to the TCGA cohort. **(F)** the ICGC cohort.

We analyzed the relationship between the model and clinicopathological features of HCC patients. As shown in **Figure 3A**, age, gender, race, prior malignancy, and radiation therapy were not statistically meaningful for the prediction of prognosis. Otherwise, CRG scores were related to pathologic stage in the TCGA cohort and ICGC cohort (**Figures S2A, B**). On the other hand, the high score of the CRG model was an independent risk factor of low OS according to the clinical dates from TCGA (HR = 1.5267, 95%CI = 1.0144-2.298, p = 0.04253) (**Figure 3A**) and ICGC-LIRI(JP) (HR = 3.2132, 95%CI = 1.5607-6.6155, p = 0.00153) (**Figure 3B**). A nomogram was created to better quantify the predicted value of the risk model for individual HCC patients (**Figures 3C, D**), and the calibration curves showed the stability and accuracy of the risk model in the training and test cohorts (**Figures 3E, F**). The C-index was 0.66 in the TCGA group and 0.76 in ICGC-LIRI(JP) group.

### The gene signature was closely related to a variety of metabolic characteristics of HCC

**Figure 4:**
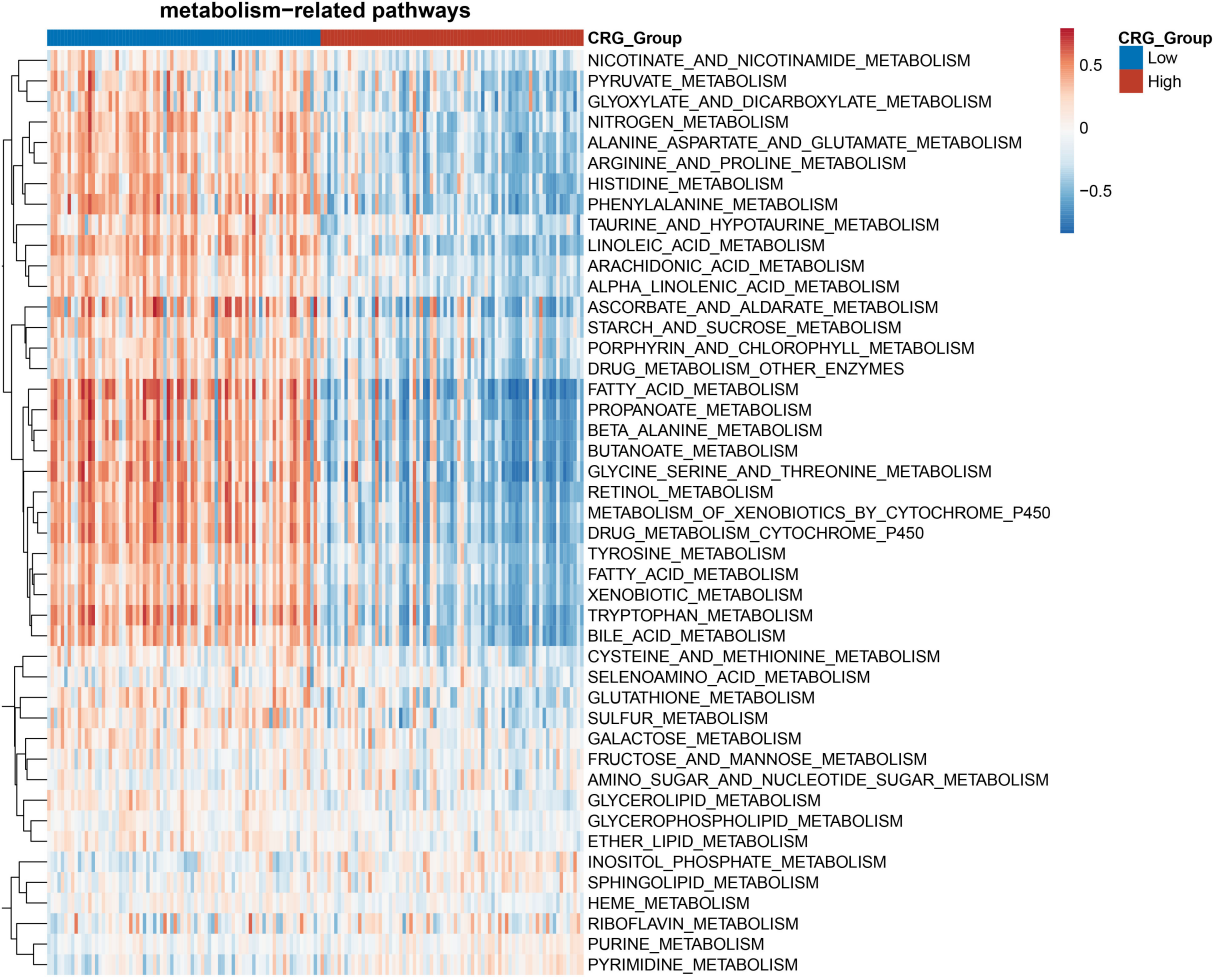
Heatmap of the enrichment analysis of metabolism-related pathways in the HCC patients with high- and low- CRG scores.

Cuproptosis is characterized by the aggregation of lipoylated proteins, which were involved in the process of the TCA cycle occurring in the mitochondria (6). The link between cuproptosis and mitochondrial proteins indicated that the effect of CRG risk score on metabolism in HCC needs to be further clarified. Gene set enrichment analysis (GSEA) based on the TCGA dataset for the two groups showed that HCC patients in the low-CRG group had enrichments in metabolism-related pathways such as pyruvate metabolism, fatty acid metabolism, alanine-aspartate-glutamate metabolism or glutathione metabolism (**Figure 4**). Specifically, Glutamine is considered as the second important nutrient only to glucose in cancer. Its metabolism begins with the conversion to glutamate by GLS (15). The glutamate could be transferred into mitochondria, transformed to alpha-ketoglutarate and participated in the TCA cycle. As for glutathione (GSH), despite being produced exclusively in the cytosol with the utilization of cysteine, glutamate or glycine, GSH is also abundant in many organelles, including peroxisomes, the nucleus, endoplasmic reticulum and mitochondria (16). The enrichment analysis confirmed that the metabolism-related changes of high-CRG HCC patients centralized in pyrimidine metabolism or purine metabolism. The pyrimidine metabolism together with purine metabolism, final generates deoxyribonucleoside triphosphates (dNTPs) in the nucleus, cytoplasm and mitochondria for cell proliferation (17) (**Figure 4**). In our article, we only initially analyzed the varied performance of metabolism-related pathways in two groups. Metabolomics analysis of clinic samples could be used to judge the changes in metabolic substrate and for deeper exploration.

### The gene signature served as a valuable marker for immune targets and immunotherapy response

**Figure 5:**
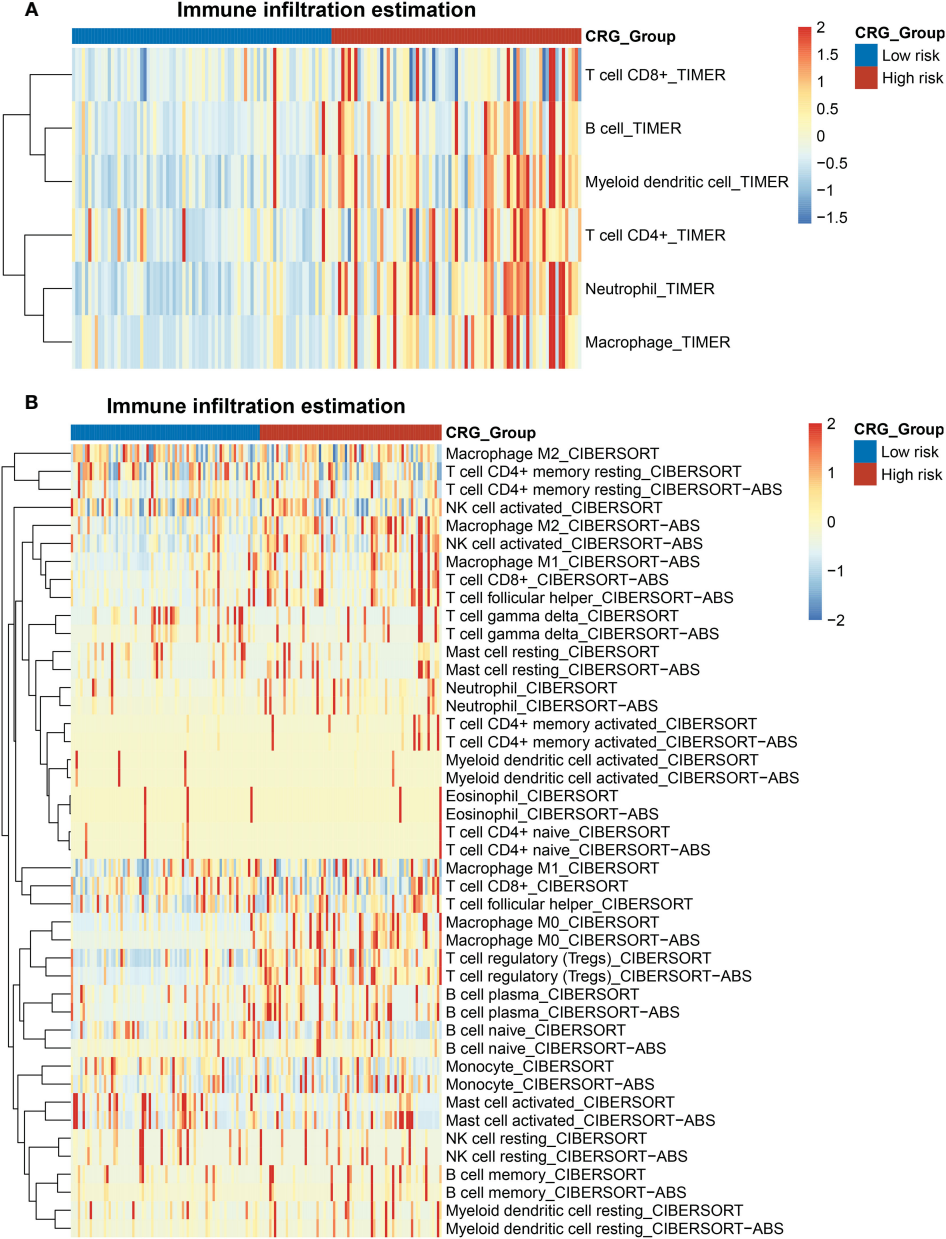
Heatmap of the enrichment scores of immune cells in the HCC patients with high- and low- CRG scores. **(A)** TIMER algorithm. **(B)** CIBERSORT and CIBERSORT-ABS algorithm.

Tumor immunology plays a key role in HCC development and progression (18). We evaluated the immune landscape of CRG and found that high-CRG patients had significantly higher immune scores than low-CRG patients. Besides, using the TIMER algorithm, we initially noticed different scores of macrophages, B cells, CD4+ T cells, and neutrophils in two groups (**Figure 5A**). According to the results of the CIBERSORT and CIBERSORT-ABS algorithm, more specific features were presented in subtypes of immune cells, such as macrophage M0, M1, and M2 (**Figure 5B**). Immune infiltration scores of macrophages M0 were higher in the high-risk group in the CIBERSORT, while the other two subtypes showed no statistically significant difference between the two groups. Based on CIBERSORT-ABS, all three subtypes in the high-risk group were higher than the low-risk group (p < 0.01) (**Figures S3A, B**). We performed an ssGSEA analysis to observe immune cell subpopulations, immune functions, and pathways in the high-risk and low-risk groups or risk scores. The results showed that the immune cells including macrophages myeloid DC, Tregs cell, neutrophil and CD4+ Th2 cell were markedly expressed in the high-risk group of the TCGA cohort (p < 0.05) (**Figure S4**). Regarding immune function, the risk levels of lipid mediators, glycogen metabolism, glucose deprivation and TCA cycle were higher in the low-risk group than in the high-risk group, when the functionality of the G1/S and G2/M was opposite to these functions (**Figure S5**). Immune checkpoints in high- and low-CRG groups relying on TCGA dataset were found to be different, such as BN2A1, BTN2A2, CD276, CD47, CD70, and HLA all highly expressed in high-CRG groups (**Figure S6A**). Aa for PD-L1, the expression of PD-L1 of high-CRG group was higher in both datasets (**Figures S6B, C**), which proved the correlation between PD-L1 and cuproptosis.

**Figure 6:**
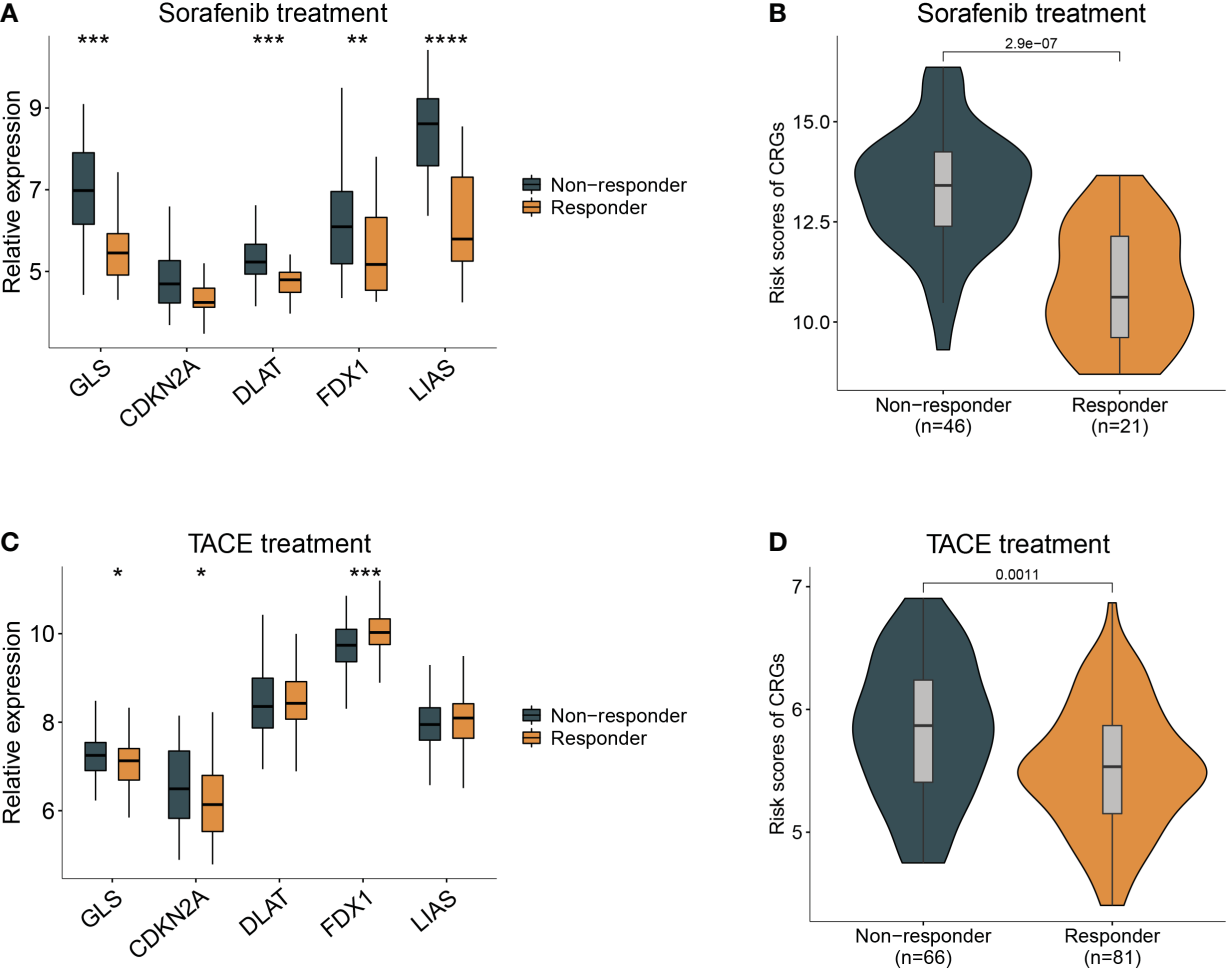
Guidance of CRG score in the therapy for HCC patients. **(A)** The different responses to sorafenib in the HCC patients with different expression of cuproptosis-related genes. **(B)**, with high- and low- CRG scores. **(C)** The different responses to TACE treatment in the HCC patients with different expression of cuproptosis-related genes. **(D)**, with high- and low- CRG scores. Wilcoxon test was used for data analyses.

Sorafenib worked as the first-line treatment for advanced-stage HCC patients (19). Therefore, we evaluated the effect of sorafenib for different CRG risk patients and found that the response of sorafenib treatment followed the relative expression of GLS, DLAT, FDX1, and LIAS and risk scores of CRG (**Figures 6A, B**) (GSE109211). Patients with a lower score were more sensitive to sorafenib treatment (p < 0.01). Similar results were observed in the analysis for transcatheter arterial chemoembolization (TACE) treatment, a widely used therapy for HCC patients at stage BCLC A or B (20). Patients who responded to TACE treatment had significantly variations in expression levels of GLS, CDKN2A, FDX1 and higher values of CRG (p = 0.0011) (**Figures 6C, D**).

### GLS was associated with poor prognosis and cuproptosis in HCC

**Figure 7:**
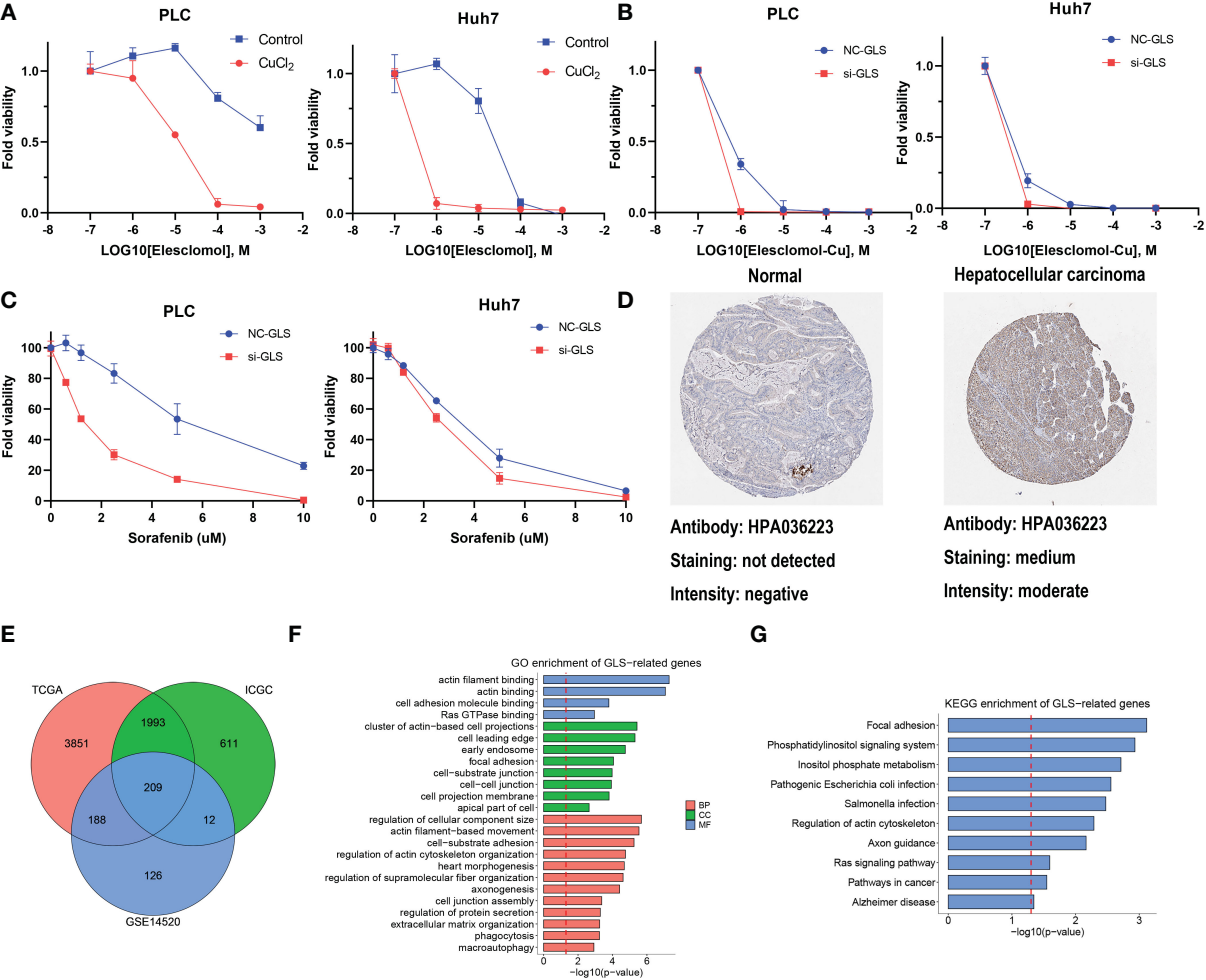
The function of GLS in HCC. **(A)** Viability of cells after treatment with elesclomol with or without 1uM of CuCl_2_. **(B)** Viability of GLS-knockdown or not cells after treatment with elesclomol with 1uM of CuCl_2_. **(C)** Viability of GLS-knockdown or not cells after treatment with sorafenib. **(D)** The protein expression levels of GLS in Human Protein Atlas database. **(E)** GLS-related genes across TCGA, ICGC and GSE datasets. **(F)** GO enrichment analysis for GLS. **(G)** KEGG pathway enrichment analysis for GLS.

GLS had the highest HR in the multivariate Cox regression analysis and droped our attention. The expression level of GLS was related to pathologic stage in the TCGA cohort (**Figure S7A**). We initially validated the function of CuCl_2_ in the HCC cell line (PLC and Huh7) related to cuproptosis *via* elesclomol. The results confirmed that the addition of Cu^2+^ enhanced the effect of elesclomol (**Figure 7A**). The decreased expression of the GLS gene reduced the cell death induced by elesclomol-Cu (**Figures 7B**, **S7B, C**). In addition, when the GLS gene was knocked down in PLC and Huh7, they were more sensitive to sorafenib (**Figure 7C**), corresponding to the previous prediction. The immunohistochemical staining for GLS highlighted its high expression in HCC tissue (**Figure 7D**). Percentage of positive of HCC group including low positive, positive, high positive, is about 61%. Percentage of positive of non-HCC group is about 24%. After systemically analyzing three databases (**Figure 7E**), GO analysis showed that they were enriched in actin binding, Ras GTPase binding and so on (**Figure 7F**). Meanwhile, KEGG analysis indicated that GLS was related to Ras signaling pathway (**Figure 7G**). In summary, *in vitro*, experimental data suggest that high expression of GLS is related to cuproptosis and poor prognosis in patients with HCC.

## Discussion

Traditionally, copper was recognized as an active site cofactor to mediate a variety of essential cellular functions, such as antioxidant defense, biosynthesis of hormones, pigments, neurotransmitters and mitochondrial respiration (21–23). This has been challenged by its newly-discovered role of dynamic signaling metal and metalloallosteric regulator that promote copper-dependent cell proliferation (cuproplasia) and copper-dependent cell death (cuproptosis) (24). According to recent studies, cuproptosis was characterized by the aggregation of mitochondrial lipoylated proteins and the destabilization of Fe–S cluster proteins triggered by copper (6). The liver is a major site of copper storage and removal. Some studies have demonstrated the role of copper in tumorigenesis and the development of HCC (25, 26). The increased incidence of HCC in patients with Wilson disease highlights the unknown mechanism of copper-related malignant transformation (27). In addition, previous studies have established the correlation between serum copper level and overall survival or other prognostic indicators (28, 29). Although decades of studies highlight the interplay between copper and HCC, a systemic molecular explanation of copper does not perform from present researches, and few studies have provided insights into the role of cuproptosis in HCC progression.

Here, we obtained 27 cuproptosis-related genes from a previous study with a FDR score <0.05. The LASSO regression detected that only five (FDX1, GLS, CDKN2A, DLAT, and LIAS) possessed compatible prognostic values considering their expression level and molecular function. The differential expression gene analysis of the TCGA dataset identified all five candidate genes, and FDX1, encoding a small iron-sulfur protein, was recently found to regulate dihydrolipoamide S-acetyltransferase (DLAT) lipoylation and facilitates the oligomerization of lipoylated DLAT (5). The above process attributed to copper-related cell death in a completely different way from apoptosis, pyroptosis, necroptosis, or ferroptosis. GLS encodes a K-type mitochondrial glutaminase, which mainly participates in glutamine metabolism and glutathione (GSH) production (30). GLS could regulate stemness properties of cancer stem cells by increasing ROS accumulation and suppressing the Wnt/β-catenin pathway in HCC (31). CDKN2A (cyclin-dependent kinase inhibitor 2A) is related to three alternatively spliced variants: two kinds of CDK4 kinase or a stabilizer of the tumor suppressor protein p53 (32). CDKN2A was reported to be a tumor suppressor gene. However, it is a highly-expressed gene associated with poor prognosis and immune infiltration in HCC patients (33). As a member of the radical S-adenosylmethionine (SAM), human lipoyl synthase (LIAS) is an enzyme containing two [4Fe–4S] clusters involved in the biosynthesis of the lipoyl cofactor (34). By lipoylation of E2 subunit (dihydrolipoyl succinyltransferase [DLST]) within the mitochondria, LIAS was involved in tricarboxylic acid (TCA) cycle process (35). In addition, the results of the single-cell sequence confirmed that FDX1, GLS, and CDKN2A were predominantly detected in malignant HCC cells, suggesting the involvement of cuproptosis-related genes in the development of HCC.

In the risk genes model, the high-CRG group showed a considerably shorter survival time, and the model performed satisfactorily for prognostic prediction in the training and validation cohorts. The external validation of our model in clinical settings revealed that the prognostic risk score model was an independent prognostic indicator. In addition, a risk-assessment nomogram was used to evaluate the potential clinical application of this model.

We focused on the metabolic and immunological pathways to establish a biological landscape of two groups divided by our five-genes model. The immunologic and metabolic pathways play a leading role in tumorigenesis of HCC, and was chosen as targets of therapeutic researches. Cuproptosis relied on the mitochondrial TCA cycle and was influenced by oxygen concentration (5). The metabolism−related pathways analysis corroborated with previous results. The low-risk group showed enrichments in mitochondrial metabolism and aerobic glycolysis, such as pyruvate and glutathione metabolism. Otherwise, the low-risk group involved the enrichment of fatty acid metabolism, bile acid metabolism, and amino acid metabolism like alanine, arginine, serine, and glycine, which might be related to oxidative stress. The results of immune infiltration estimation showed the macrophage scores varied in the two groups. The resting macrophages (M0) could be polarized into different phenotypes, pro-inflammatory (M1) or anti-inflammatory (M2) (36). M1 were reported to potentially participate in antitumor immunity. Conversely, M2 could enhance tumor progression by promoting angiogenesis, fibrosis, immunosuppression, lymphocyte exclusion, invasion, and metastasis (37). Nevertheless, how cuproptosis or cuproptosis-inducing drugs affect the function of antitumor immune cells and the metabolic changes remains unclear.

GLS was known as a mitochondrial glutaminase, increasing glutathione and glutamine levels. In a recent study, treatment of HCC cell lines (HCCLM3, SMMC-7721, and Hep3B) with GLS1 inhibitors halted the growth of HCC cell lines (38). As shown in the “Cuproptosis” article, GSH inhibited cuproptosis *via* copper(I), where Chung et al. established the relevance of copper and glutathione in cancer cells. Their work revealed that oncogene-driven changes in the metabolism of glutathione lead to a labile copper(I) deficiency, insinuating that lower GSH/GSSG ratios decreased labile copper(I) availability but did not affect total copper level (39). The theory suggests that copper(I) ion, which is essential for DLAT aggregation and cuproptosis, would prefer forming complexes with “S-ligands” of GSH and protect proteins from copper-induced damage might explain the inhabitation of GSH for cuproptosis (40). In addition, Zuily and collaborators reported that copper toxicity is related to copper-induced protein aggregation, and treatment with copper(I) under anaerobic conditions leads to severe ROS-independent protein aggregation (41). The knockdown of GLS was associated with the exacerbation of copper toxicity with elesclomol. This evidence highlights the role of GLS in HCC copper-related cell death.

Some limitations existed in our study. First, the construction and validation of the prognostic model were based on retrospective public datasets, thus prospective studies are required to verify the accuracy and utility of our model. Second, some environmental and genetic factors closely related to the occurrence of HCC are inevitably missing. Finally, more *in vivo* and *in vitro* experiments regarding the relationships between cuproptosis -related genes and HCC should be further performed.

## Conclusion

In conlusion, a novel CRG, based on cuproptosis-related genes, was constructed using the LASSO Cox regression model. HCC patients with a high CRG scores were revealed to be associated with shorter survival time, lower enrichment in metabolic-related pathways, and high infiltration scores of protumor immune cells. In our model, among all included CRG genes, GLS was marked as a functional gene in the development of HCC, and might be involved in the cuproptosis process in HCC.

## Data availability statement

The datasets presented in this study can be found in online repositories. The names of the repository/repositories and accession number(s) can be found in the article/supplementary material.

## Author contributions

(i) Conception and design: BL and JL. (ii) Administrative support: BL and JL. (ii) Experiments implementation: DP and NW. (iii) Data analysis and bioinformatics analysis: GN and YW. (iv) Manuscript writing: GN. (v) Final approval of manuscript: All authors. All authors contributed to the article and approved the submitted version.
