# Supplementary material for: Cuproptosis-related genes score: A predictor for hepatocellular carcinoma prognosis, immunotherapy efficacy, and metabolic reprogramming
Source: Front Oncol. 2023 Feb 9;13:1096351. doi: 10.3389/fonc.2023.1096351 (PMC9947795; doi:10.3389/fonc.2023.1096351)
Supplement: Supplementary file 1 [file DataSheet_1.pdf]

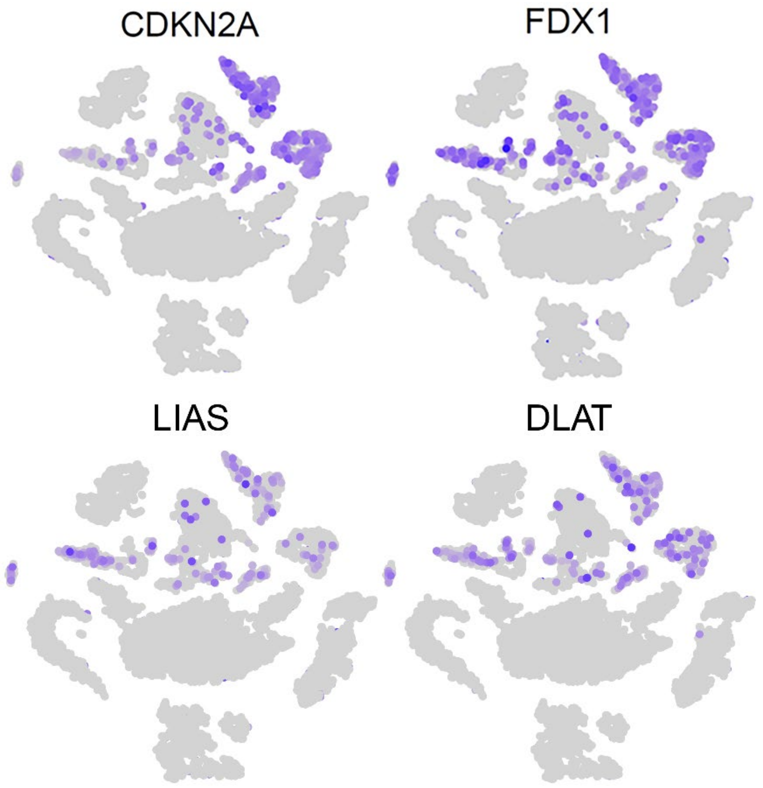

Figure S1. The expression of CRG-included genes in eight types of cells in the GSE125449 dataset.

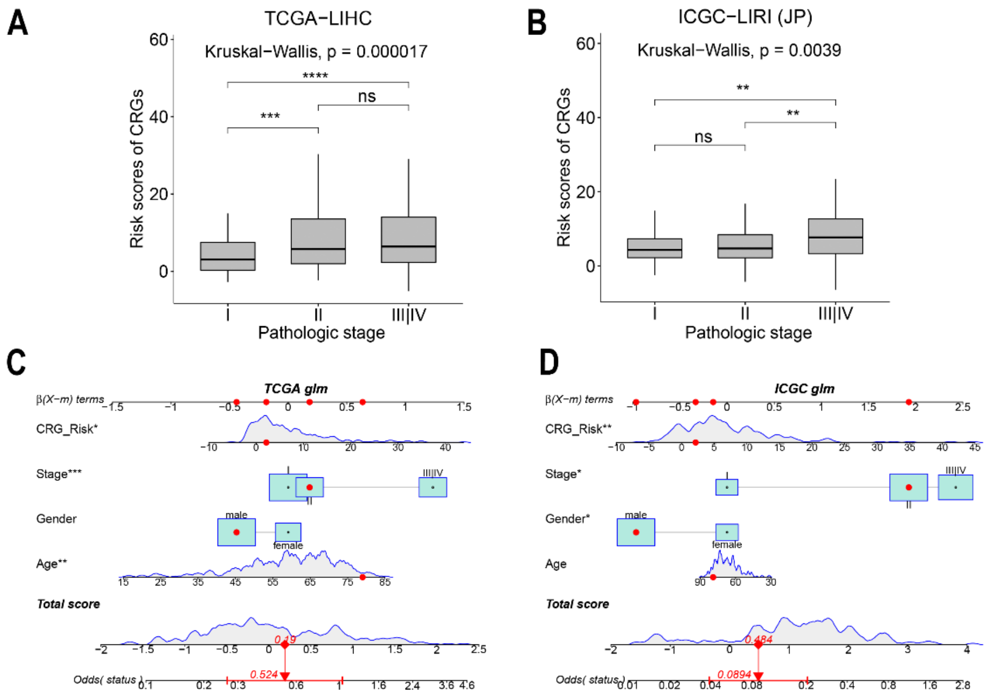

Figure S2. Establishment and validation of the model via nomogram. (A) CRG scores were related to pathologic stage in the TCGA cohort. (B), in the ICGC cohort. (C) Nomogram plot via GLM in the TCGA cohort. (D), in the ICGC cohort.

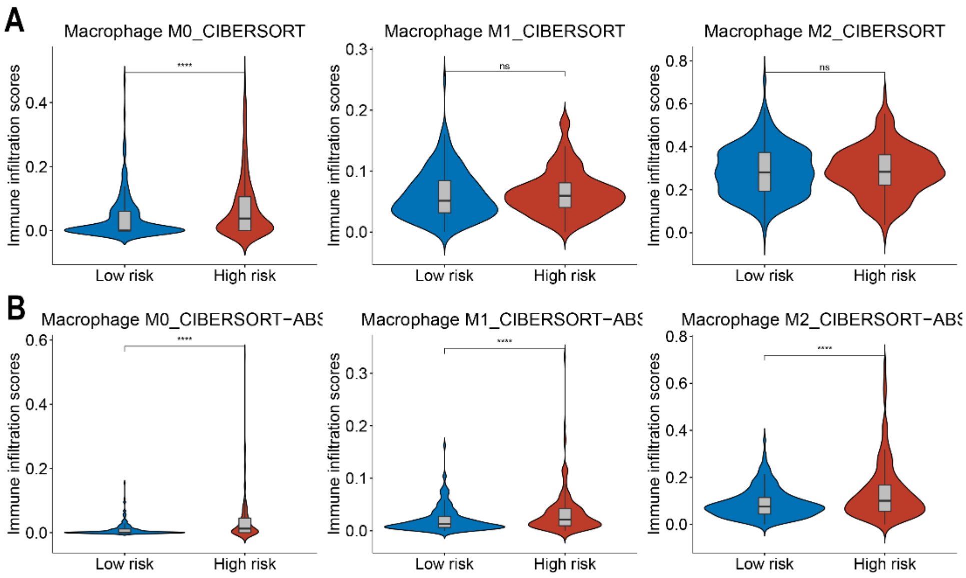

Figure S3. Different macrophage M0, M1, and M2 scores in the high- and low- CRG groups via (A) CIBERSORT and (B) CIBERSORT-ABS algorithm.

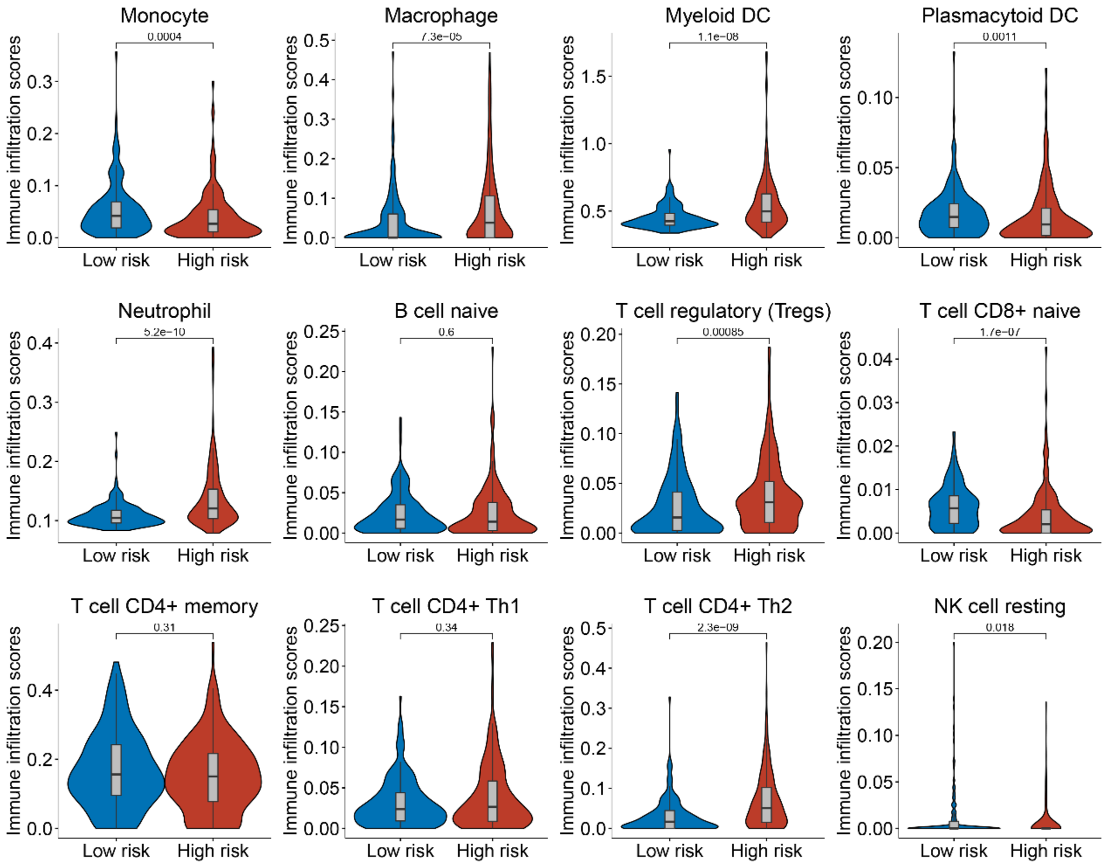

Figure S4. The scores of 16 immune cells were detected by ssGSEA analysis based on different risk groups in TCGA cohort.

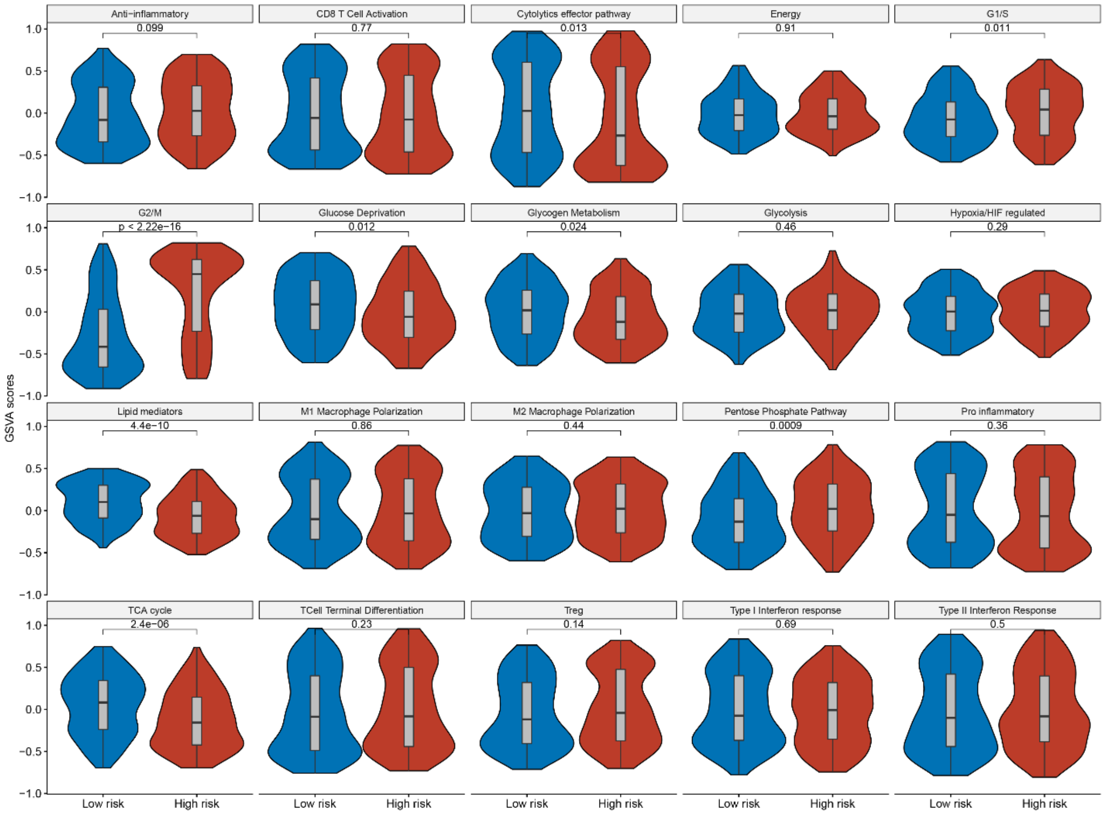

Figure S5. The scores of 20 immune-related functions were detected by ssGSEA analysis based on different risk groups in TCGA cohort.

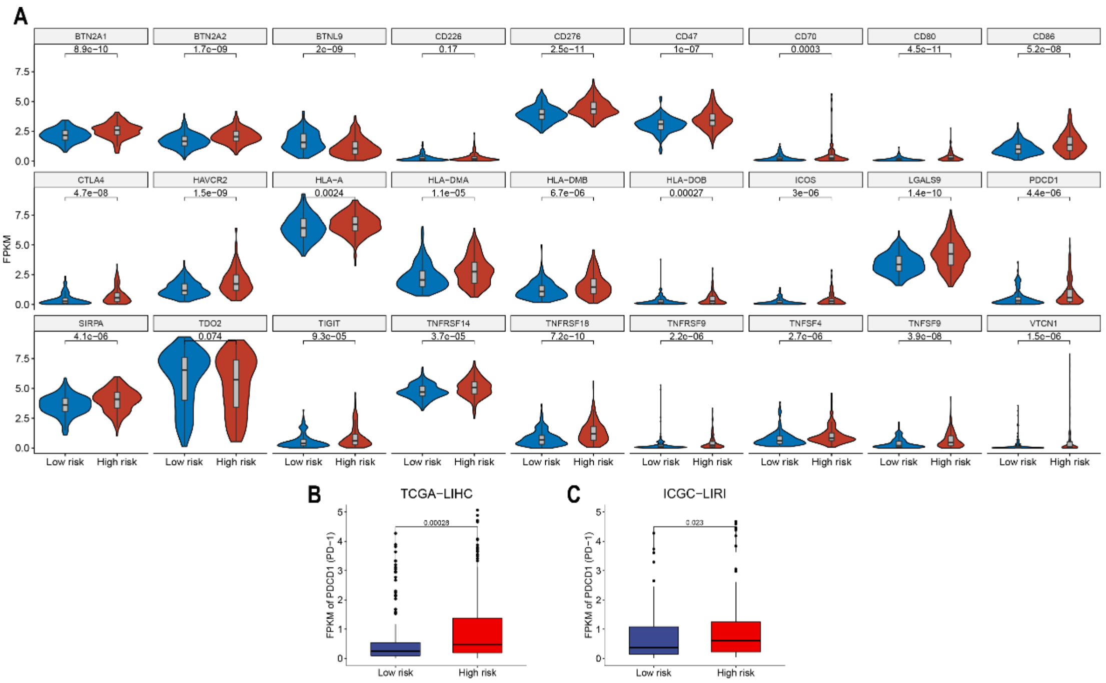

Figure S6. Correlation between the signature and the immune-related genes. (A)The expression of immune checkpoints in high- and low-CRG groups in TCGA dataset. (B)The expression of PD-L1 in high- and low-CRG groups in TCGA dataset. (C)in ICGC dataset.

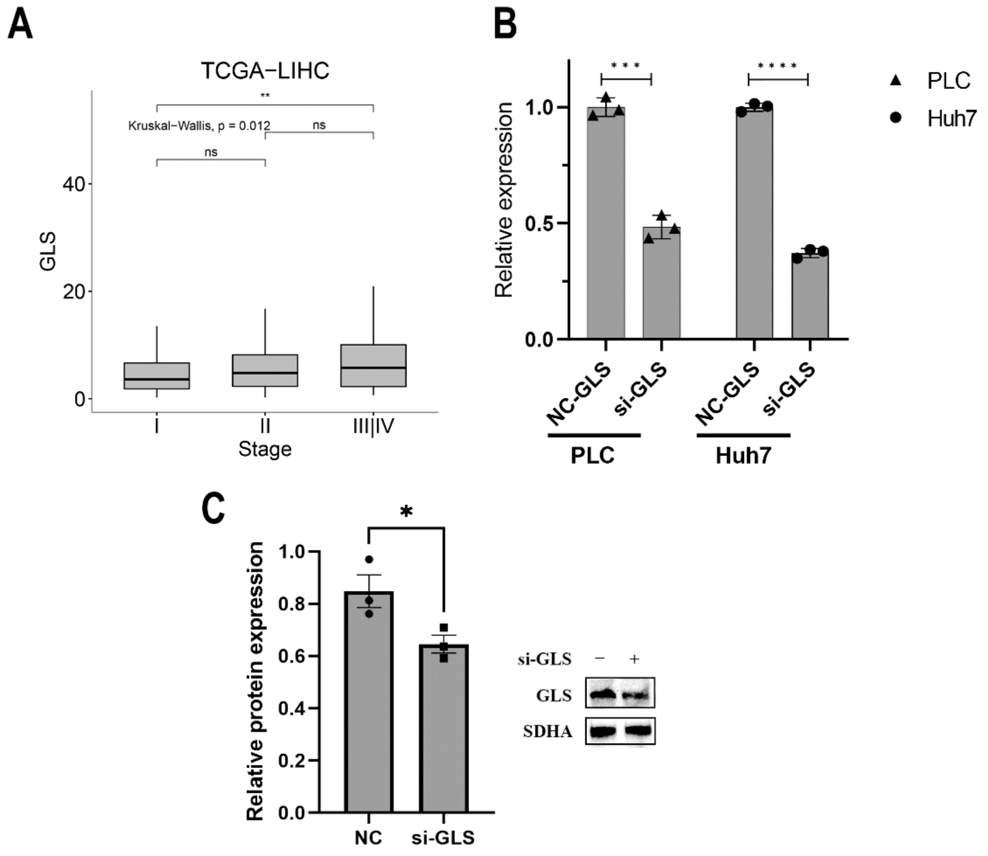

Figure S7. The function of GLS in HCC. (A) The expression level of GLS was related to pathologic stage in the TCGA cohort. (B) The knockdown efficiency of si-GLS. （C）The protein expression of GLS after knockdown (PLC cell line). Data were expressed as mean ± SEM.
